# Clinical application of a new latex photometric immunoassay reagent, LPIA‐GENESIS D‐dimer, and its performance in patient‐derived plasma samples

**DOI:** 10.1111/ijlh.13169

**Published:** 2020-02-29

**Authors:** Yutaka Nagahama, Junko Nozaki, George Sakurai, Satoshi Yajima, Noriko Kaneko, Etsuko Yamazaki, Michio Matsuda

**Affiliations:** ^1^ IVD Business Segment LSI Medience Corporation Tokyo Japan; ^2^ Clinical Laboratory Department Yokohama City University Hospital Yokohama Japan; ^3^ Jichi Medical University Tochigi‐ken Japan

**Keywords:** antibody for D‐dimer, blood collection, cross‐linked fibrin degradation products (XDP), D‐dimer, latex photometric immunoassay (LPIA)

## Abstract

**Introduction:**

We previously reported an antibody MIF‐220 that recognizes a specific structure induced on the surface of thrombin‐activated E‐domain of one fibrin molecule bound with the D‐domains of other fibrinogen/fibrin molecules. Utilizing MIF‐220, we produced a test kit for cross‐linked fibrin degradation products (XDP), LPIA‐GENESIS D‐dimer (LG‐DD), and evaluated basic performance characteristics for clinical application. We then attempted to apply LG‐DD to see its eligibility in clinical plasma samples.

**Method:**

The characteristic performances requested for clinical use were studied including limit of quantitation, within‐run imprecision, day‐to‐day imprecision, antigen excess, interference study, and method comparison with LPIAACE‐Ddimer (ACE‐DD) available on the market.

**Results:**

The performance characteristics were all satisfactory. Extraordinarily high concentrations of XDP are occasionally obtained by ACE‐DD in samples with collection problems, but not by LG‐DD, indicating that a certain XDP species present in the former was not measured by LG‐DD. Structural studies suggested that the “B‐b” set of polymerization sites must be involved as well in the maintenance of cross‐linked fibrin in vivo.

**Conclusion:**

LG‐DD was able to measure a wide range of XDP, that is, 0.20‐35.0 μg FEU/mL that covers the levels of XDP in most of the clinical samples. LG‐DD was found to almost avoid false‐positive results noticed in samples as mentioned above, and this feature seems to be preferable to established kits for the measurement of XDP.

## 1 INTRODUCTION

We previously reported a monoclonal antibody raised against a single component DD/E derived from plasmic digests of human cross‐linked fibrin (XDP) designated as (DD/E)_n_, where “n” stands for positive numbers.^1^ This antibody was able to recognize a specific structure induced on the surface of thrombin‐activated E‐domain of one fibrin molecule bound with the D‐domains of other fibrinogen/fibrin molecules or with the isolated DD‐domains derived from XDP schematically shown as (D‐E‐D) and (DD/E), respectively. This antibody was named as “MIF‐220.”

As anticipated, MIF‐220 failed to react with fibrinogen or its degradation products by plasmin (FgDP) without prior activation by thrombin. When the thrombin‐activated human fibrin E‐domain was allowed to react with the D_1_‐domains derived from other animal species such as ovine and bovine fibrinogens, reaction proceeded to form molecular complexes D_1_‐E‐D_1_ or D_1_‐E that are both recognized by this antibody. On the contrary, the antibody was unable to react with the thrombin‐activated E‐domains of these animal species bound with the human D_1_‐domains, although the molecular complexes had been formed. Thus, it appears that the binding sites for this antibody are induced cryptically on the E‐domain of human molecule and become available when the E‐domain of fibrin molecule is bound with the D‐domains of fibrinogen/fibrin molecules. The binding capacity of this antibody for (D‐E‐D) was, however, markedly reduced in the absence of calcium ions. On the contrary, the binding capability for (DD/E) containing the cross‐linked DD structure was unaffected regardless of the presence or the absence of calcium ions. It appears that certain structures represented by the calcium‐stabilized conformation of the D‐domain and/or the factor XIIIa‐mediated cross‐linking between two D‐domains are required for recognition of the putative binding site by this antibody.

Using MIF‐220, we prepared a latex photometric immunoassay reagent for the measurement of XDP and tested the performance characteristics requested for its clinical application. This reagent named LPIA‐GENESIS D‐dimer (LG‐DD) has been available in Japan on the market and will come into the market in other countries shortly.

As the data on XDP measured with LG‐DD in clinical samples have been accumulated in Japan, considerable discrepancies have occasionally been reported on the levels of XDP as compared with those measured with established kits available on the market. Namely substantially increased concentrations of XDP are observed in blood samples obtained with collection problems, mostly spending too much time for collection of blood samples from the patients. Interestingly such increases are not observed in the samples obtained without collection problems from the same patients at certain intervals.

In this study, we attempted to analyze the molecular basis for the discrepancies to be attributed to secondary activation of blood coagulation and fibrinolysis that may have occurred in samples with collection problems.

## 2 MATERIALS AND METHODS

### 2.1 Fibrinogen‐related antigens

Fibrinogen‐related antigens were obtained from commercial sources: human fibrinogen (Enzyme Research Laboratories): bovine thrombin (Mochida Pharmaceutical): human plasmin (CALBIOCHEM): Aprotinin and Reptilase (Pentapharm).

Plasmic degradation products of fibrinogen were prepared as described by Nieuwenhuizen et al for fragments X and Y^2^, ^3^ and by van Ruijven‐Vermeer et al for fragment D_1_.^4^ XDP species were prepared as described by Olexa and Budzynski.^5^ The XDP preparations were subjected to Sephacryl S‐300 (GE Healthcare) gel chromatography and divided into two fractions, as represented by DD/E, that is, [(DD/E)_n_, n = 1] and [(DD/E)_n_, n ≥ 2]. Of these two fractions, the [(DD/E)_n_, n ≥ 2] fraction was used for XDP standard material for the assay.

Fibrin clots formed with either thrombin or Reptilase were solubilized by 20 mmol/L acetic acid, and they were expressed as desAABB‐fibrin monomer (desAABB‐FM) and desAA‐fibrin monomer (desAA‐FM), respectively. Soluble Fibrin monomer‐fibrinogen complex (SF) was prepared as described by Soe et al.^6^

### 2.2 Antibodies

JIF‐23,^7^ a monoclonal antibody that recognizes the amino‐terminal region of fragment D_1_, and IF‐43,^6^ a monoclonal antibody that recognizes a neo‐antigen exposed in the E domain of FM complexed with fibrinogen or its derivatives and MIF‐220, were our own products.

### 2.3 Reagent kits and the auto‐analyzer

For the measurement of XDP, our newly developed product: LG‐DD utilizing MIF‐220 was used on a fully automated clinical assay system LPIA‐STACIA (LSI Medience) together with ACE‐DD utilizing JIF‐23 from LSI Medience. XDP was measured with Nanopia D‐dimer (SEKISUI MEDICAL) and LIAS AUTO D‐dimer NEO (Sysmex) as well. An aliquot of pooled human serum (Golden West Diagnostics) was treated with JIF‐23 to remove XDP if any, and used as a diluent for test plasma samples (diluent‐A).

Plasmin‐α2 plasmin inhibitor complex (PPI) and SF were measured on LPIA‐STACIA as well, using LPIAACE PPI II and IATRO SF II (both LSI Medience), respectively. For the measurement of APTT, SynthASil APTT and ACL TOP System (Instrumentation Laboratory) were used. Normal ranges of these parameters were shown as follows, APTT: 25.0 to 35.0 seconds, XDP: <0.5 μg FEU/mL, PPI: <0.8 μg/mL and SF: <7 μg/mL, respectively.

### 2.4 Clinical samples

Clinical plasma samples subjected to our study were obtained from Yokohama City University Hospital under the approval by the Human Ethics Review Committees of Yokohama City University Hospital.

Citrated plasmas derived from 315 patients were enlisted to this study. These samples were selected out of the samples tested for blood coagulation and fibrinolysis to detect the disseminated intravascular coagulation syndrome (DIC), deep vein thrombosis (DVT) and/or pulmonary embolism, and also for the management of the patients after operation.

Samples with collection problems: When we noted shortening of APTT (<24.0 seconds) or the presence of visible fibrin clots in 59 samples, we found that they had been separated from samples with collection problems as observed in approximately 0.2% in our laboratory. Therefore, we attempted to collect another samples from the patients without delay as reference. These samples were called the first and the second samples, respectively. Another 256 samples were obtained without any collection problems. Plasmas were stored at −80°C until use.

### 2.5 Performance characteristics of LG‐DD

#### 2.5.1 Limit of quantitation (LOQ)

We prepared a sample containing 0.35 μg FEU/mL XDP in diluent‐A, and progressively diluted the sample with diluent‐A at a difference of 0.035 μg FEU/mL. Ten solutions containing XDP from 0.35 μg FEU/mL down to 0.035 μg FEU/mL were thus prepared. We quantitated XDP in these samples in duplicate for five consecutive days. The concentration of XDP which gave a coefficient of variation (CV) of 10% was defined as LOQ.

#### 2.5.2 Within‐run imprecision

Three different concentrations of XDP, that is, low, middle, and high of the measurable range, were quantitated ten times, and CV’s were calculated.

#### 2.5.3 Day‐to‐day imprecision

Three different concentrations of XDP were quantitated once a day for 5 days, and CV’s were calculated.

#### 2.5.4 Linearity

We prepared a sample containing 58 μg FEU/mL XDP in diluent‐A, and progressively diluted the sample with diluent‐A at a difference of 5.8 μg FEU/mL. Ten solutions containing XDP from 58 μg FEU/mL down to 5.8 μg FEU/mL were thus prepared. A sample containing 5.8 μg FEU/mL XDP was further diluted by serial twofold dilution up to 32‐folds giving 0.18 μg FEU/mL. Prepared 15 samples were measured with LG‐DD.

#### 2.5.5 Antigen Excess

To study the recognizable antigen‐excess range of XDP for LG‐DD, we prepared various concentrations of XDP by serial twofold dilution up to 32‐folds with diluent‐A containing 255 μg FEU/mL that was the maximum value experienced in clinical laboratories. Prepared samples were measured with LG‐DD.

#### 2.5.6 Interference studies

Hemoglobin, bilirubin, and serum containing rheumatoid factor were purchased from Sysmex. INTRAFAT was purchased from Nihon Pharmaceuticals for interference study of lipaemic plasma. The triglyceride levels were measured with IATROLQ TGII (LSI Medience). Heparin sodium from porcine intestinal mucosa was purchased from Wako Pure Chemical. SF was our own product. Two samples, containing 5.8 or 23.2 μg FEU/mL XDP, spiked with interference substances, were subjected to the measurement of XDP with LG‐DD (abbreviated as XDP‐LG hereafter).

### 2.6 Evaluation of LG‐DD on clinical samples

#### 2.6.1 Method comparison

Concentrations of XDP in these 256 plasmas were measured with LG‐DD, and ACE‐DD as reference (abbreviated as XDP‐ACE hereafter). In this study, Passing‐Bablok regression was used.

#### 2.6.2 Comparison of the concentration of XDP in the first and the second samples

XDP‐LG and XDP‐ACE in 59 paired plasma samples, that is, the first and the second plasma samples, were measured. The sample with the highest XDP‐ACE in the first sample was selected for further structure analysis. The first sample and the second sample of this patient were called sample‐1 and sample‐2, respectively.

### 2.7 Molecular analysis of XDP in clinical samples recognized by LG‐DD

Briefly, 0.5 mL of the plasma sample was subjected to a Superose 6 column (GE Healthcare), eluted at 1 mL/min, and fractionated for every 0.5 minutes. The original plasma and each separated fraction were subjected to SDS‐PAGE using 3%‐8% Tris‐acetate gel under nonreducing conditions, or using 4%‐12% Bis‐Tris gel under reducing conditions. These gels were purchased from Thermo Fisher Scientific. Migrated proteins in the gel were transferred to Polyvinylidene difluoride membrane, and visualized with various polyclonal antibodies against human fibrinogen or its component fractions. They included antibodies raised in rabbits against human fibrinogen (Agilent Technologies), fibrinogen β‐chain (Proteintech Group), and fibrinogen E‐domain (our own product).

By searching for immune‐stained spots found only in sample‐1, we extracted proteins therefrom and analyzed amino acids in the first three N‐terminal cycles, and then adapted them to the most probable amino acid sequence of human fibrinogen subunits.

### 2.8 Reactivity of MIF‐220 to desAABB‐FM‐D_1_ complex and desAA‐FM‐D_1_ complex

One volume of 1 mg/mL desAABB‐FM or desAA‐FM in 20 mmol/L acetic acid was added to 9 volumes of 10 mg/mL fragment D_1_ in 50 mmol/L Tris‐buffered saline, pH 8.0 (TBS) at a molar ratio of acid‐FM: fragment D_1_ to bring 1:30, and the mixture was allowed to react for 30 minutes at 37°C. Formation of the desAABB‐FM‐D_1_ or desAA‐FM‐D_1_ complexes via the E‐D binding was confirmed with IATRO SF II, using IF‐43. Reactivity of MIF‐220 to each of these complexes was examined by an ELISA. As a control, a mixture of one volume of 20 mmol/L acetic acid and nine volumes of the D_1_ solution was used.

Fifty micro litre of 5 μg/mL MIF‐220 in TBS was pipetted into wells of polystyrene micro‐titer plate (Corning Incorporated) and allowed to stand overnight at 4°C. The wells coated with MIF‐220 were washed three times with 0.15 mol/L NaCl containing 0.05% Tween‐20. DesAABB‐FM‐D_1_ or desAA‐FM‐D_1_ complexes containing 50 μL of 10 μg/mL FM was added to MIF‐220‐coated well, and incubated for 90 minutes at 37°C. Binding of fibrin‐related antigens was monitored by a chromogenic system utilizing peroxidase‐labeled anti‐human‐fibrinogen polyclonal antibody (Agilent Technologies).

## 3 RESULTS

### 3.1 Performance characteristics of LG‐DD

#### 3.1.1 LOQ

LOQ was calculated to be 0.20 μg FEU/mL.

#### 3.1.2 Within‐run imprecision

CV’s of the assay were 3.14%, 0.21%, and 0.43% at 0.63, 9.55, and 24.6 μg FEU/mL of the standard XDP solution, respectively.

#### 3.1.3 Day‐to‐day imprecision

CV’s of the assay were 2.14%, 0.32%, and 0.81% at 0.82, 3.40, and 9.90 μg FEU/mL of the standard XDP solution, respectively.

#### 3.1.4 Linearity

The linearity was confirmed from 0.18 to 35.0 μg FEU/mL, since the per cent recovery rate defined as the measured value over the theoretical value multiplied by 100 all fell in 100 ± 10% at the 15 points we had measured.

#### 3.1.5 Antigen excess

Antigen excess was not found up to 255 μg FEU/mL.

#### 3.1.6 Interference

No interference was observed up to 30 mg/dL for bilirubin, 500 mg/dL for hemoglobin, 678 mg/dL triglyceride for lipaemia, 250 IU/mL for rheumatoid factor, 150 U/mL for unfractionated heparin, and 360 μg/mL for SF, respectively.

#### 3.1.7 Method comparison of LG‐DD with ACE‐DD

**Figure 1:**
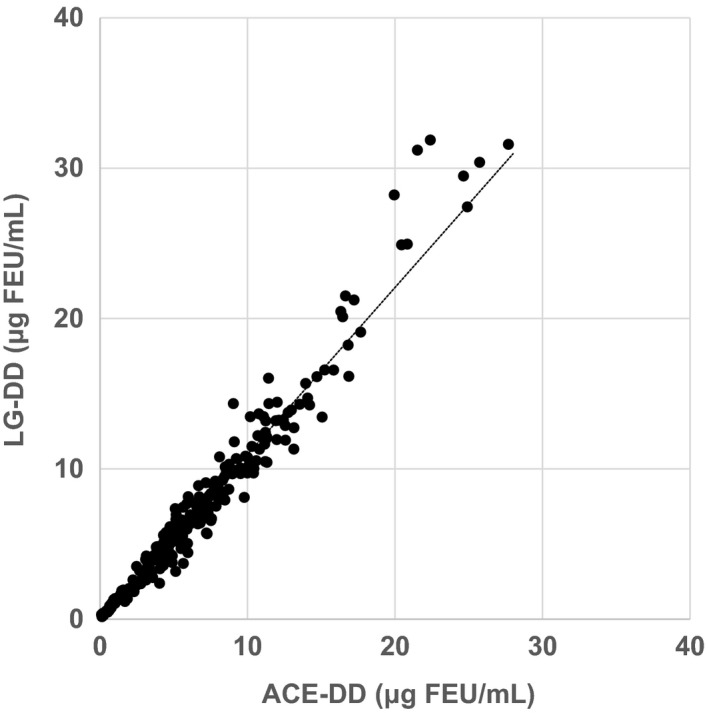
Method comparison of LG‐DD (y) with ACE‐DD (x). The regression line was y = 1.11x‐0.15, and the correlation coefficient was 0.98 (N = 256). At the lower range below 2.0 μg FEU/mL, the regression line was y = 1.04x + 0.06 and the correlation coefficient was 0.93 (N = 35)

XDP‐ACE and XDP‐LG were fairly well correlated with each other (Figure 1). The regression line was y = 1.11x‐0.15 and the correlation coefficient (*r*) was 0.98. If we focus on the lower range below 2.0 μg FEU/mL representing 35 samples, the regression line was y = 1.04x + 0.06, *r* = 0.93.

#### 3.1.8 Concentrations of XDP in the first and the second samples

**Figure 2:**
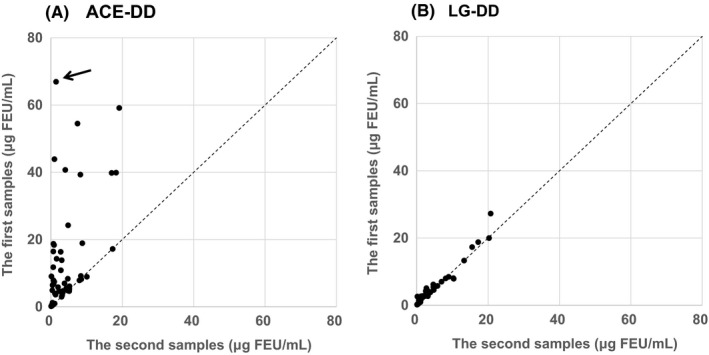
Comparison of XDP concentrations in the first and the second samples derived from 59 patients measured with ACE‐DD and LG‐DD. The first samples: Fifty‐nine samples manifesting shortening of APTT (<24.0 seconds) or the presence of visible fibrin clots were subjected to XDP measurement. The second samples: Samples were obtained without collection problems. XDP was measured by ACE‐DD (panel A) and LG‐DD (panel B) in these paired samples. The sample indicated by an arrow in panel A was chosen for analysis of marked discrepancy between XDP concentrations in the first sample and that in the second sample

Both XDP‐ACE (Figure 2A) and XDP‐LG (Figure 2B) were compared in 59 paired plasmas. Levels of XDP‐ACE in the first sample group were widely distributed up to 70 μg FEU/mL, mostly higher than those in the second sample group (Figure 2A), whereas no distinct discrepancies were noted in those of XDP‐LG in both groups (Figure 2B). The regression line of XDP‐ACE (x) and XDP‐LG (y) in the second samples was y = 0.96x + 0.01 (*r* = 0.99). When ACE‐DD was replaced with other commercially available kits, the concentrations of XDP in the first samples were mostly higher than those in the second samples as observed with ACE‐DD.

In order to see the molecular basis for this difference, we selected the patient whose first sample gave the highest XDP‐ACE. Profiles of blood coagulation and fibrinolysis studies including XDP obtained with other commercially available kits are presented in Table 1. As anticipated, concentrations of XDP measured with 4 XDP kits are well coincided with each other in sample‐2.

**Table 1:** Profiles of blood coagulation and fibrinolysis studies conducted on the selected patient

	APTT	[XDP] ACE‐DD	[XDP] LG‐DD	SF	PPI	[XDP] Nanopia D‐dimer	[XDP] LIAS AUTO D‐dimer NEO
Normal range	25.0‐35.0 (s)	<0.5 (μg FEU/mL)	<0.5 (μg FEU/mL)	<7 (μg/mL)	<0.8 (μg/mL)	<0.5 (μg FEU/mL)	<0.5 (μg FEU/mL)
Sample‐1	25.6	66.9	3.9	242	52.7	113	118
Sample‐2	34.1	1.5	1.7	1.3	2.3	1.7	1.6

Abbreviations: APTT, activated partial thromboplastin time; XDP, cross‐linked fibrin degradation products; PPI, plasmin‐α2 plasmin inhibitor complex; SF, soluble fibrin monomer‐fibrinogen complex.

XDP was measured with following kits, ACE‐DD: LPIAACE D‐DimerⅡ, LG‐DD: LPIA‐GENESIS D‐dimer, Nanopia D‐dimer, and LIAS AUTO D‐dimer NEO.

Sample‐1: sample with collection problems.

Sample‐2: sample without collection problems.

### 3.2 Analysis of fibrinogen‐related protein fragments recognized by ACE‐DD but not by LG‐DD

**Figure 3:**
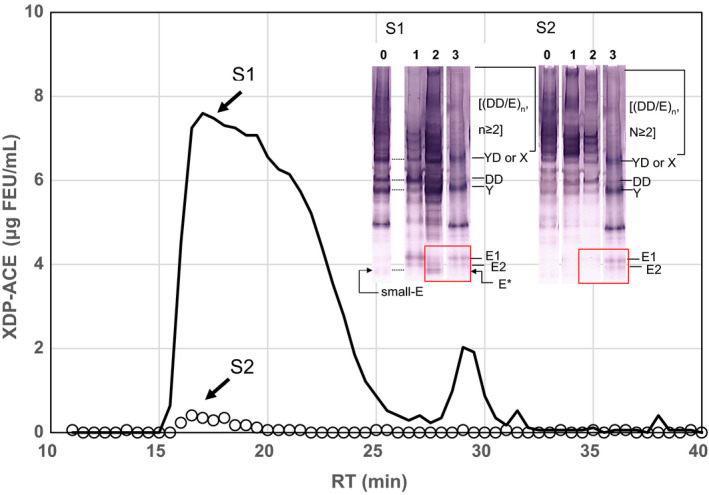
Gel filtration chromatogram on a Superose 6 column and nonreducing SDS‐PAGE followed by immunoblotting with an antifibrinogen antibody of the patient plasmas. Sample‐1 and sample‐2 were applied onto a Superose 6 column and eluates were monitored with ACE‐DD. S1: sample‐1 and S2: sample‐2. lane 0: samples applied, lane 1: RT = 17.0 min, lane 2: RT = 29.5 min and lane 3: standardized DD/E. Small‐E present in lane 0 of S1 is absent in lane 0 of S2. Band E* present in lane 2 of S1 is absent in lane 2 of S2

Sample‐1 and sample‐2 were subjected to SDS‐PAGE under nonreducing conditions. Immunoblot analysis of these two plasmas revealed that fragments X or YD, DD, Y, and D_1_ were more clearly stained for sample‐1 as compared with those for sample‐2 (lanes 0 in S1 and S2, Figure 3). There was a faint band below the two positions for E_1_ and E_2_ that was visualized by an anti‐human fibrinogen E antibody, and we tentatively called this band “small‐E.”

#### 3.2.1 Separation of fibrinogen‐related proteins by gel exclusion chromatography on Superose 6 and their SDS‐PAGE profiles

Sample‐1 and sample‐2 were subjected to Superose 6 column, and XDP‐LG and XDP‐ACE were measured in each fraction. The retention time (RT) on this column for [(DD/E)_n_, n ≥ 2], fibrinogen, DD/E, and D_1_ are 16, 23, 28, and 33.5 minutes, respectively. Fractions with RT = 17.0 and RT = 29.5 minutes for these samples were subjected to immunoblotting. Chromatograms of XDP‐ACE are shown for sample‐1 and sample‐2 (Figure 3). Chromatographic profiles of XDP‐LG were quite similar with one another (profiles not shown). Furthermore, these chromatograms resembled the profile observed in sample‐2 as monitored by ACE‐DD.

Immunoblotting studies run under non‐reducing conditions for RT = 17.0 and 29.5 minutes fractions of sample‐1 are shown in lanes 1 and 2 (S1 in Figure 3), and those of sample‐2 are shown in lanes 1 and 2 (S2 in Figure 3). There is an additional band designated as E* in the RT = 29.5 minutes fraction which migrated faster than the bands E_1_ and E_2_.

**Figure 4:**
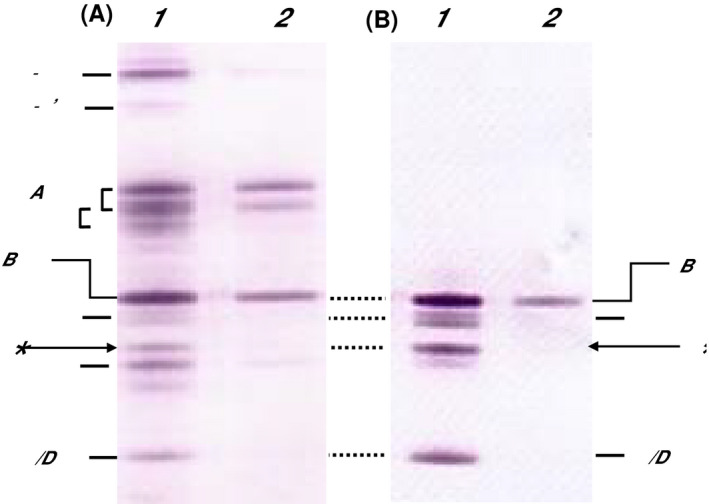
SDS‐PAGE under reducing conditions followed by immunoblot analysis of the fractions eluted at 17.0 min of the sample‐1 and sample‐2. Sample‐1 and sample‐2 were applied onto a Superose 6 column. By chromatogram monitored with ACE‐DD, the peak fractions eluted at 17.0 min were subjected to SDS‐PAGE under reducing conditions followed by immunoblot analysis. A, visualized with an anti‐fibrinogen antibody. B, visualized with an anti‐fibrinogen β‐chain antibody. Lanes 1: sample‐1 and lanes 2: sample‐2. The bands indicated by an asterisk in lanes 1 are absent in lanes 2

Immunoblotting was run under reducing conditions for respective RT = 17.0 minutes fractions of sample‐1 and sample‐2, and separated proteins were visualized with anti‐fibrinogen antibody (Figure 4A) and with anti‐fibrinogen β‐chain antibody (Figure 4B). To be noted here is the presence of a protein band indicated by an asterisk in sample‐1 stained with both antibodies.

When we extracted small‐E in the sample applied and analyzed amino acids in the first three N‐terminal cycles, we found at least three peptides with the first three amino acids consisting of Asp‐Leu‐Gly, Asp‐Leu‐Gln, and Lys‐Val‐Glu.

### 3.3 Reactivity of MIF‐220 to desAABB‐FM‐D_1_ and desAA‐FM‐D_1_ complexes

MIF‐220 reacted with desAABB‐FM‐D_1_ but not with desAA‐FM‐D_1_.

## 4 DISCUSSION

LOQ, within‐run imprecision, day‐to‐day imprecision, linearity, antigen excess, and interference studies were found to be satisfactory. Deducing from the data, the range of XDP measured by LG‐DD was 0.20 to 35.0 μg FEU/mL. The same characteristic performances were obtained in clinical plasma samples as well, as reported elsewhere.^8^, ^9^, ^10^

When XDP‐LG and XDP‐ACE were compared, they were fairly well correlated with each other, especially, in a low XDP range below 2.0 μg FEU/mL including the cut‐off value for DVT exclusion, 0.5 μg FEU/mL. In higher XDP ranges, XDP‐LG were higher than XDP‐ACE in some samples. As we know that LG‐DD is more sensitive to smaller components of XDP than ACE‐DD as reported previously,^8^ elevated levels of XDP may reflect that small‐sized XDP species are predominant due to increased fibrinolysis in the patients.

As shown in Figure 2, XDP‐ACE in the first samples were higher than those in the second samples, whereas no distinct discrepancy was noted in XDP‐LG. In clinical laboratories, we occasionally encounter falsely elevated XDP due to activation of coagulation and fibrinolysis that has taken place in syringes. In such cases, we have to collect plasma samples again and report revised data to clinical wards. Therefore, LG‐DD may contribute to establish appropriate judgement and management of disease conditions that are not necessarily achieved by other commercially available kits.

The results prompted us to see whether some molecular masses recognized by ACE‐DD but not by LG‐DD were present in sample‐1. By immunoblot analysis of these two samples run under non‐reducing conditions, we found a fibrinogen‐related protein fragment, small‐E (lane 0 in S1, Figure 3).

Sample‐1 and sample‐2 were subjected to Superose 6 column (Figure 3). For sample‐1, there were two major fractions on the chromatogram, where the first broad peak was found to be a group of [(DD/E)_n_, n ≥ 2] and another peak fraction eluted at 29.5 minutes was apparently composed of large varieties of protein fragments as judged by SDS‐PAGE visualized by an anti‐fibrinogen antibody. Besides bands E_1_ and E_2_ derived from DD/E, an additional protein band E* was positively visualized with an anti‐human E antibody (lane2 in S1, Figure 3).

In sample‐2, there was a broad peak eluted around at 16‐27 minutes corresponding to [(DD/E)_n_, n ≥ 2], whereas no distinctly measurable proteins by ACE‐DD were noted in the vicinity of 29.5 minutes (open circles, Figure 3). Since proteins in the standardized sample of DD/E was eluted at 28 minutes on the same column, the peak fraction eluted at 29.5 minutes was considered to be composed of fibrinogen‐related proteins smaller than DD/E.

We identified at least three peptides in the sample applied with the first three amino acids consisting of Asp‐Leu‐Gly, Asp‐Leu‐Gln, and Lys‐Val‐Glu, and assigned them to the Bβ (71‐73), the γ (39‐41), and the Bβ (54‐56) segments, respectively.

Considering increased fibrinolysis that may have occurred in the sample during the blood sample collection as noted by extraordinarily increased XDP‐ACE, the subunit protein with its N‐terminal Bβ (54‐56) may well be attributed to the Bβ (54‐121) segment cleaved by plasmin at positions Lys53‐Lys54 and Lys121‐Asp122.

These results indicate that the band small‐E is most likely to be derived from the E domain that lacks the B‐polymerization site, that is, the Bβ (15‐17) segment on the N‐terminus of its β‐chains. The bands, small‐E, and E* seem to be identical as judged by SDS‐PAGE profiles and reactivity to an antifibrinogen‐E antibody.

Concerning a broad and large peak composed of fractions with the retention time from 16 to 27 minutes of sample‐1, protein compositions could be representatively shown by [(DD/E)_n_, n ≥ 2], where “n” decreased in accordance with the retention time. Immunoblotting of the proteins eluted at 17 minutes separated by SDS‐PAGE run under nonreducing conditions revealed that the fibrinogen/fibrin‐related proteins in this fraction were mostly [(DD/E)_n_, n ≥ 2], fragments X or YD, DD and Y (lane 1 in S1, Figure 3). To see the profiles of subunits of these fragments that must have been modified by thrombin and plasmin, we attempted to analyze the proteins by immunoblotting after SDS‐PAGE run under reducing conditions. When the resolved proteins were visualized with an antifibrinogen antibody, there were multiple bands corresponding to γ‐γ, γ‐γ’, Aα, α, Bβ, β, γ, and β/D, all of which had been derived most likely from the cross‐linked fibrin, SF and its degraded products.

Furthermore there was an additional band indicated by an asterisk between β and γ (lanes 1, Figure 4). This band was named “short‐β” as it was positively visualized with an antifibrinogen‐β antibody. In the fraction eluted at 17 minutes of sample‐2 obtained without collection problems, solely fibrinogen‐derived subunits were observed (lanes 2, Figure 4). Since the short‐β was present in sample‐1 but not in sample‐2, we allotted this protein to the Bβ (54‐461) segment that lacks the Bβ (1‐53) segment due to cleavage of the Bβ‐chain by plasmin. Indeed, the electrophoretic mobility of this protein seems to well coincide with this β‐chain segment consisting of 408 amino acid residues.

These findings may also indicate that the presence of the B‐polymerization site is a prerequisite for the recognition of XDP by LG‐DD, and that XDP further digested by plasmin lacking the B‐polymerization site would not be detected by LG‐DD.

We then prepared desAA‐FM by treating fibrinogen with a snake‐venom, Reptilase, to expose only the A‐sites and allowed the desAA‐FM to bind with fragment D_1_. As anticipated, MIF‐220‐immobilized ELISA failed to recognize the complex of desAA‐FM with D_1_, although MIF‐220 was able to recognize the complex of desAABB‐FM with D_1_.

The data altogether seem to support the notion that the set of B‐b polymerization sites participates in the formation of stable fibrin, as propose by Yang et al.^11^ Furthermore, the cross‐linked fibrin degradation products represented by [(DD/E)_n_, n ≥ 1] in clinical plasma samples appear to be mostly, if not all, desAABB‐FM complexed with D_1_‐containing fragments of fibrinogen and fibrin, as shown in Figure 1. This finding may be supported by reports from several laboratories.^8^, ^9^, ^10^ The XDP further digested by plasmin may thus appear under some special conditions as observed in the blood samples with collection problems.

Based on lines of information, we have obtained heretofore together with other studies published elsewhere on the structures of cross‐linked fibrin molecules and their degradation products,^12^, ^13^ we propose models of soluble molecular complexes of XDP with special reference to the participation of two sets of polymerization sites, “A‐a” and “B‐b,” for the expression of the epitope for MIF‐220. For full expression of the epitope, participation of both polymerization sites “A” and “B” exposed on the E‐domain as well as the cross‐linking formed between the D‐domains of two fibrin molecules appear to be mandatory. Removal by plasmin of the “B” site exposed on the N‐terminal segment of the β‐chain seems to destroy the structure required for recognition by this antibody.

## 5 CONCLUSION

We prepared a test kit, LG‐DD, for the measurement of XDP on an autoanalyzer, and attempted to evaluate this test kit for the measurement of XDP in plasma samples derived from the patients manifesting abnormalities in blood coagulation and fibrinolysis studies.

Regarding the requirements for the performance characteristics including LOQ, within‐run imprecision, day‐to‐day imprecision, linearity are all confirmed to be satisfactory. No interference by bilirubin, hemoglobin, lipaemia, rheumatoid factor or heparin was observed. LG‐DD was able to measure a wide range of XDP, that is, 0.20 to 35.0 μg FEU/mL that covers the levels of XDP in most of the clinical samples.

These characteristic features of LG‐DD are expected to selectively pick up XDP in plasma samples by avoiding SF‐related macromolecular complexes and XDP lacking the B‐b binding generated in syringes during blood collection as occasionally encountered upon the use of commercially available test kits for XDP. The feature of LG‐DD will reduce the probability to report false positive result to doctors.

In the last, we would like to emphasize that the antibody, MIF‐220, may also contribute to further studies on fibrin‐related macromolecular complexes initiated by specific binding of “A‐a” followed by “B‐b” and factor XIIIa‐mediated cross‐linking formed between fibrin molecules.

## CONFLICT OF INTEREST

SY, NK, and EY received grants as co‐operative research fund from LSI Medience Corporation. MM received a payment as an advisor from LSI Medience Corporation.

## AUTHOR’S CONTRIBUTIONS

YN, JN, and GS designed the experiments, performed the experiments, and wrote the manuscript. SY, NK, and EY performed the experiments. MM designed the experiments and wrote the manuscript. All authors read, revised, and approved the final version of the manuscript.
